# Peripheral blood B lymphocytes derived from patients with idiopathic pulmonary arterial hypertension express a different RNA pattern compared with healthy controls: a cross sectional study

**DOI:** 10.1186/1465-9921-9-20

**Published:** 2008-02-12

**Authors:** Silvia Ulrich, Laima Taraseviciene-Stewart, Lars C Huber, Rudolf Speich, Norbert Voelkel

**Affiliations:** 1 Department of Internal Medicine, Pulmonary Hypertension Clinic, University Hospital of Zurich, Zurich, Switzerland; 2 Department of Medicine, Pulmonary Sciences and Critical Care Medicine, University of Colorado Health Sciences Centre, Denver, Colorado, USA

## Abstract

**Background:**

Idiopathic pulmonary arterial hypertension (IPAH) is a progressive and still incurable disease. Research of IPAH-pathogenesis is complicated by the lack of a direct access to the involved tissue, the human pulmonary vasculature. Various auto-antibodies have been described in the blood of patients with IPAH. The purpose of the present work was therefore to comparatively analyze peripheral blood B lymphocyte RNA expression characteristics in IPAH and healthy controls.

**Methods:**

Patients were diagnosed having IPAH according to WHO (mean pulmonary arterial pressure ≥ 25 mmHg, pulmonary capillary occlusion pressure ≤ 15 mmHg, absence of another explaining disease). Peripheral blood B-lymphocytes of patients and controls were immediately separated by density gradient centrifugation and magnetic beads for CD19. RNA was thereafter extracted and analyzed by the use of a high sensitivity gene chip (Affymetrix HG-U133-Plus2) able to analyze 47000 transcripts and variants of human genes. The array data were analyzed by two different softwares, and up-and down-regulations were defined as at least 1.3 fold with standard deviations smaller than fold-changes.

**Results:**

Highly purified B-cells of 5 patients with IPAH (mean pulmonary artery pressure 51 ± 13 mmHg) and 5 controls were analyzed. Using the two different analyzing methods we found 225 respectively 128 transcripts which were up-regulated (1.3–30.7 fold) in IPAH compared with healthy controls. Combining both methods, there were 33 overlapping up-regulated transcripts and no down-regulated B-cell transcripts.

**Conclusion:**

Patients with IPAH have a distinct RNA expression profile of their peripheral blood B-lymphocytes compared to healthy controls with some clearly up-regulated genes. Our finding suggests that in IPAH patients B cells are activated.

## Introduction

Idiopathic pulmonary arterial hypertension (IPAH) is histopathologically characterized by endothelial cell proliferation and formation of plexiform lesions. Plexiform lesions are often found to be surrounded by immune cells, which have been identified as B- and T-lymphocytes, macrophages and mast cells [1,2]. The possible pathogenetic role of the immune system in IPAH is further supported by the close association between various immune disorders and pulmonary arterial hypertension and the frequent finding of autoimmune antibodies and altered cytokine status in serum of IPAH patients [3,4]. B-lymphocytes (B-cells) are fundamental for the humoral immune response due to their potential to differentiate into antibody-producing plasma B-cells. But B-cells also play a crucial role in cell-mediated immune regulation through antigen presentation, production of various cytokines, differentiation of T effector cells and collaboration with antigen-presenting dendritic cells and lymphoid organogenesis. Antibodies directed against pulmonary endothelial cells have been found in IPAH[5]. The generation of these autoantibodies from mature B-cells might be explained by a different RNA expression profile between B-cells of patients with IPAH and healthy control cells and thus might present a different translational functionality. Moreover, the differential RNA-pattern of B-cells in IPAH might provide helpful information to elucidate the pathogenetic role of the immune system in IPAH and might be of diagnostic value in the early detection of the disease.

## Methods

### Subjects

Patients were diagnosed with IPAH according to WHO if the mean pulmonary artery pressure was ≥ 25 mmHg by right heart catheterization and an extensive clinical work-up did not reveal other conditions responsible for pulmonary arterial hypertension[6]. Patients and healthy volunteers gave their written informed consent and the study was approved by the local institutional review board (Colorado Multiple Institution Review Board [COMIRB]).

### Blood collection and B cell separation and RNA-extraction

10 ml of peripheral blood was collected in tubes containing ethylenediamineteraacetic acid and samples were processed within 30–60' after blood drawn under careful and frequent decontamination of the working space and all materials needed (RNAseZAP, Ambion, TX, US, Cat #9790). The blood was diluted in three volumes of phosphate-buffered saline + 2-mM ethylenediamineteraacetic acid + 0.5% bovine serum albumin. The peripheral blood mononuclear cell (PBMC) layer was isolated via density gradient centrifugation (Histopaque 1077, Sigma-Aldrich, St. Louis, USA), at 1200 rpm for 30 min. B cells were magnetically separated from PBMC's using MACS anti-CD19 micro beads (Miltenyi Biotec, Bergisch-Gladbach, Germany). Purity of the B-cell separation was assessed by flow-cytometry after staining 100'000 cells with fluorescently labelled monoclonal antibody against CD 19 (anti-CD19-APC respectively FACS Calibur, BD Biosciences, NY, USA). B-cell pellets were dissolved in 1 ml of TRI reagent (Ambion, Tx, US, Cat) and stored at -80°C until RNA extraction.

All frozen B cells samples were simultaneously defrosted on ice. After thawing, the RNA was extracted using RiboPureTM-Kit (Ambion, Tx, US Cat # 1924) according to the manufacture's instruction and stored at -20°C until RNA microarray was performed.

### Microarray data generation

RNA quality assessment, sample preparation, reverse transcription, labeling, high-density oligonucleotide array hybridization, scanning and data analysis were performed according to standard practice [7-10]. Samples were analyzed by the use of the Affymetrix HG-U133-Plus2 gene chip, which is able to analyze 47,000 transcripts and variants, including 38,500 well-characterized human genes due to its high resolution (Affymetrix, CA, US). Fluorescence intensities were quantified using the affymetrix Microarray Analysis Suite 5.0 (MAS5) and Robust Multichip Analysis (RMA) statistical algorithm with default parameters for the array type used in this study (Affymetrix HG-U133-Plus2, CA, US).

### Data analysis and statistics

Detailed protocols for data analysis of Affymetrix microarrays and extensive documentation of the sensitivity and quantitative aspects of the method have been described[11]. In brief, the array data were analyzed by GeneChip^® ^Operating Software (GCOS, Affymetrix, CA, US) and genesprings software (GSS, Agilent Technologies, CA, US). Both softwares are able to statistically analyze quantitative signal expression levels retrieved from the Affymetrix microarray with GCOS mainly used for comparison of expression profiles between single patients across groups and GSS used to compare differential expression profiles between groups (e.g. healthy vs diseased). The raw data from array scans were averaged across all gene probes on each array by MAS5 and RMA, two different mathematical algorithms to process, background-correct and normalize raw data from microarray gene chips, thereafter, a scaling factor was applied to bring the average intensity for all probes on the array to 500. For further normalization of the raw data all signal values were log transformed (log base 2), values below 0.01 were set to 0.01, each measurement was divided by the 50.0^th ^percentile of all measurements in that sample and each gene was divided by the median of its measurements in all samples. To define up- and down-regulated genes in IPAH versus healthy controls, all genes whose flags were present or marginal in all 5 IPAH samples (for up-regulated genes) or not-present (for down-regulated genes) in comparison with controls were selected as first filter technique by GSS. As second filter, up- and down regulated genes were selected if they were present or marginal in 4 of the 5 IPAH versus control samples and if a statistical difference was present between IPAH and control values (Student's t-test, p-value cut-off 0.05). By using GSS, all genes with normalized data values >1.3 fold higher respectively lower than in the other groups were selected. By our second software, the GCOS, we select all genes which were up- or down-regulated in IPAH vs. controls in at least 15 of the possible 25 comparisons with a fold change (FC) greater than the calculated standard deviation for each gene.

## Results

**Figure 1:**
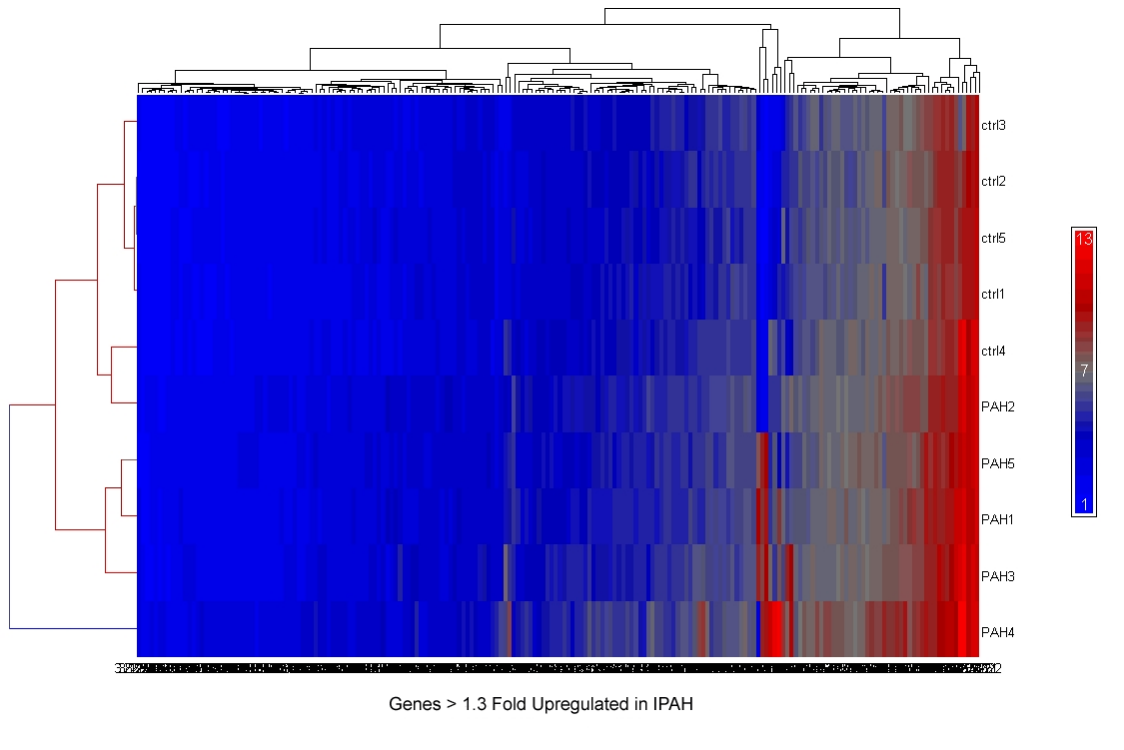
Cluster dendrogram of up-regulated genes in 5 patients with idiopathic pulmonary arterial hypertension (lower rows, PAH-number) and 5 healthy controls (upper rows, ctrl-number). The color-scale goes from blue (not up-regulated) to red (highly up-regulated) as indicated (scale on the right).

B-cells from five Caucasian patients with severe IPAH (mean age 46 ± 8.1 years, 3 females, mean pulmonary artery pressure 51 ± 13 mmHg, mean cardiac index 2.2 ± 0.2 ml/min/m^-2^) and five healthy Caucasian controls (mean age 47 ± 8.7 years, 3 females) were analyzed. Four of the patients were on intravenous epoprostenol therapy. The B-cell purity checked by flow cytometry detecting fluorescently labeled CD-19 was > 97%. B-cell RNA and raw data quality was good as per the specialist in the GeneChip processing core facility of the university of Colorado Health Science Centre (UCHSC). Using GCOS we found 225 genes which were at least 1.3 fold up-regulated in IPAH vs. controls (1.3 – 30.7 fold, SD < FC). Using GSS we found 128 up-regulated genes (1.3–178 fold). Combining analysis by both methods, we found overlap of 33 up-regulated genes (table 1, figure 1). Of interest, many of the up-regulated genes belong to biological processes involved in inflammation and immune responses, suggesting the activation of B cells in patients with IPAH In contrast, we found no down-regulated genes.

**Table 1:** Up-regulated genes in IPAH vs. controls: at least 1.3 fold up-regulated in GCOS and GSS

**Gene Name**	**Fold Change**	**Description**	**Gene Symbol**	**GO Biological Process Description**
205033_s_at	30.76	defensin, alpha 1, myeloid-related sequence	DEFA1 /// DEFA3	xenobiotic metabolism /// response to virus /// defense response to bacteria /// defense response to fungi /// defense response /// response to pest, pathogen or parasite
204351_at	15.05	S100 calcium binding protein P	S100P	endothelial cell migration
232629_at	10.03	prokineticin 2	PROK2	activation of MAPK activity /// angiogenesis /// anti-apoptosis /// chemotaxis /// inflammatory response /// elevation of cytosolic calcium ion concentration /// neuropeptide signaling pathway /// spermatogenesis /// cell proliferation /// sensory percept
202917_s_at	5.678	S100 calcium binding protein A8 (calgranulin A)	S100A8	inflammatory response
211654_x_at	5.479	major histocompatibility complex, class II, DQ beta 1	HLA-DQB1	immune response /// immune response /// antigen presentation, exogenous antigen /// antigen processing, exogenous antigen via MHC class II
209773_s_at	5.435	ribonucleotide reductase M2 polypeptide	RRM2	DNA replication /// deoxyribonucleoside diphosphate metabolism /// DNA replication
220005_at	5.189	G protein-coupled receptor 86	P2RY13	signal transduction /// G-protein coupled receptor protein signaling pathway
225987_at	4.787	likely ortholog of mouse tumor necrosis-alpha-induced adipose-related protein	STEAP4	fat cell differentiation /// electron transport
209773_s_at	5.435	ribonucleotide reductase M2 polypeptide	RRM2	DNA replication /// deoxyribonucleoside diphosphate metabolism /// DNA replication
220005_at	5.189	G protein-coupled receptor 86	P2RY13	signal transduction /// G-protein coupled receptor protein signaling pathway
225987_at	4.787	likely ortholog of mouse tumor necrosis-alpha-induced adipose-related protein	STEAP4	fat cell differentiation /// electron transport
212999_x_at	4.322	major histocompatibility complex, class II, DQ beta 1	HLA-DQB1	immune response /// immune response /// antigen presentation, exogenous antigen /// antigen processing, exogenous antigen via MHC class II
220167_s_at	3.448	TP53TG3 protein	TP53TG3	---
223204_at	3.429	hypothetical protein DKFZp434L142	DKFZp434L142	---
213975_s_at	2.807	lysozyme (renal amyloidosis)	LYZ /// LILRB1	carbohydrate metabolism /// cell wall catabolism /// cytolysis /// defense response to bacteria /// immune response /// response to virus /// tRNA aminoacylation for protein translation
209514_s_at	2.779	RAB27A, member RAS oncogene family	RAB27A	intracellular protein transport /// small GTPase mediated signal transduction /// protein transport
228898_s_at	2.13	similar to putative NADH oxidoreductase complex I subunit homolog.	SMARCB1	chromatin remodeling /// transcription /// regulation of transcription from RNA polymerase II promoter /// cell cycle /// DNA integration /// negative regulation of progression through cell cycle /// retroviral genome replication /// regulation of transcription
201310_s_at	2.039	chromosome 5 open reading frame 13	C5orf13	---
227724_at	1.956	hypothetical gene supported by AK091744	LOC439987	---
208704_x_at	1.92	amyloid beta (A4) precursor-like protein 2	APLP2	G-protein coupled receptor protein signaling pathway
222688_at	1.916	yf40c04.s1 Soares fetal liver spleen 1NFLS Homo sapiens cDNA clone IMAGE:129318 3', mRNA sequence.	PHCA	protein biosynthesis /// ceramide metabolism
214864_s_at	1.852	glyoxylate reductase/hydroxypyruvate reductase	GRHPR	L-serine biosynthesis /// excretion /// metabolism /// metabolism
230126_s_at	1.774	KIAA0876 protein	JMJD2B	regulation of transcription, DNA-dependent
207426_s_at	1.774	tumor necrosis factor (ligand) superfamily, member 4 (tax-transcriptionally activated glycoprotein 1, 34kDa)	TNFSF4	immune response /// signal transduction /// cell-cell signaling /// positive regulation of cell proliferation
212998_x_at	1.759	major histocompatibility complex, class II, DQ beta 2	HLA-DQB2	immune response /// immune response /// antigen presentation, exogenous antigen /// antigen processing, exogenous antigen via MHC class II
212495_at	1.745	KIAA0876 protein	JMJD2B	regulation of transcription, DNA-dependent
221581_s_at	1.676	Williams-Beuren syndrome chromosome region 5	LAT2	intracellular signaling cascade /// calcium-mediated signaling /// immune response /// mast cell degranulation /// B cell activation
208248_x_at	1.672	amyloid beta (A4) precursor-like protein 2	APLP2	G-protein coupled receptor protein signaling pathway
226456_at	1.66	hypothetical protein MGC24665	MGC24665	---
208703_s_at	1.651	amyloid beta (A4) precursor-like protein 2	APLP2	G-protein coupled receptor protein signaling pathway
212496_s_at	1.625	KIAA0876 protein	JMJD2B	regulation of transcription, DNA-dependent
223445_at	1.588	dystrobrevin binding protein 1	DTNBP1	organelle organization and biogenesis /// sensory perception /// visual perception /// response to stimulus
225593_at	1.578	U7 snRNP-specific Sm-like protein LSM10	LSM10	nuclear mRNA splicing, via spliceosome /// mRNA processing
223361_at	1.537	similar to HSPC280	C6orf115	---
224948_at	1.477	mitochondrial ribosomal protein S24	MRPS24	---

## Discussion

In the present study we comparatively investigated B-cell RNA expression profiles in patients with IPAH and healthy controls. We hereby found that IPAH patients slightly differed from healthy controls with some clearly up-regulated genes consistently found by two different analysis methods.

IPAH is a devastating and progressive condition of unknown etiology affecting the pulmonary circulation with a dismal prognosis [6]. Research on the pathobiology of IPAH on the molecular level is limited by a lack of a direct and early access to the site of pathology, the human lung. One research strategy therefore lays in the analysis of easily obtainable peripheral blood samples from IPAH patients in order to retrieve both information on possible underlying disease mechanisms and potential diagnostic tools. Recently, a strategy of assessing RNA-expression profiles of peripheral blood mononuclear cells by microarrays was introduced and shown to be able to differentiate variously classified pulmonary arterial hypertension patients and healthy controls[12]. In this work we focus this strategy based on microarray technology towards peripheral blood B-cells, based on the hypothesis to find specifically differential RNA expression profiles in a disease where various auto-antibodies have been found in the peripheral blood[4,5,13]. Indeed we found a slightly distinct RNA-expression profile with some up-regulated genes on the transcript level. At this point however, we can only speculate about the biological significance of these up-regulated genes and will in the following discuss some of them with potential value in respect to the pathogenesis of IPAH (table 1). Strikingly, many of the up-regulated transcripts are involved in inflammatory mechanisms, host defense or endothelial function. Human defensins are small cationic peptides involved in various biological processes associated primarily with defensive and regulatory responses to infections by pathological agents but they also have immunoregulatory properties, associated with their ability to bind and activate the G(i)-protein-coupled seven-transmembrane receptors and are chemoattractants for dendritic cells and memory T cells[14]. Increased airway epithelial defensin concentrations were found in association with various pulmonary infections[15] and plasma alpha-defensin concentrations were found increased in pulmonary sarcoidosis, a disease often associated with an until now unidentified infectious agent [16]. It is increasingly recognized that a deregulated immune system plays a pathogenetic role in IPAH[17,18], although a clear idea about an initial trigger and potentially involved pathways is still lacking. The herein found clear up-regulation of the B-cell RNA encoding for defensin alpha 1 in IPAH may indicate that a hitherto unknown infectious trigger may be pathogenetically involved. Other herein-found up-regulated transcripts associated with inflammatory mechanisms are sequences encoding for the major histocompatibility complex class II (HLA_DQB1 and 2), ribonucleotide reductase M2 polypeptides (which confer resistance to hydroxyurea in lymphoblastic and other tumor cell lines [19] and members of the tumor necrosis factor superfamily.

Other herein found up-regulated transcripts in B-cells of IPAH patients are involved in vessel biology, vasomotor regulation, angiogenesis or cell proliferation. Tumor-like proliferating endothelial and smooth muscle cell accumulating in the so called plexiform lesions are the cornerstone of histologic finding in IPAH. The S-100 calcium binding protein is not only a marker of tumor cell lines (e.g. melanoma or neurogenous tumors), it is also involved in cell proliferation and vasoconstriction[20,21]. Prokineticins, herein found 10-fold up-regulated in IPAH patient B-cells vs. healthy controls, are multifunctional secreted proteins able to activate distinct endogenous G-protein coupled pathways, thereby stimulating Ca2+ mobilization and cAMP accumulation[22]. Prokineticins also play a role in circadian rhythms [23], they also seem to have different pathophysiological roles in various endothelial cell systems[24,25]. Another potentially interesting herein found up-regulated transcript encodes for the purinergic receptor P2Y (or G-protein coupled receptor 86), which has not only been described playing a role in some leukemias and cancers[26,27], but has recently been implicated in the risk of atherothrombosis, namely ischemic stroke, myocardial infarction and venous thromboembolism[28]. This protein deserves being evaluated by future research in IPAH, the intrinsic illness of the pulmonary vasculature where microthrombosis is one of the key pathologic features. Interestingly, also transcripts encoding for different types of amyloid beta precursor-like proteins are herein consistently found up-regulated in IPAH. Amyloid beta proteins are important initiating molecules in the pathogenesis of Alzheimer's disease[29]. But the beta amyloid precursor protein is also highly expressed in the endothelium on neoforming vessels suggesting that it may play a role during angiogenesis[30]. An association between pulmonary arterial hypertension and Alzheimer's disease has not been described so far; however, autopsy studies reveal that venous thrombosis and atherosclerotic cardiovascular diseases are highly common comorbidities in Alzheimer patients, so the question of a potential association with pulmonary vascular disease as well may merit evaluation in light of our findings[31].

Our study has several limitations: our sample size investigating 5 patients and controls each is rather small, however, it included a broad and costly gene chip in order to retrieve the highest amount of possibly involved genes. Another limitation of our study is that we do not know if changes in the peripheral blood B-cell RNA expression profiles found are related to, cause or consequence of the pressure elevation found in the pulmonary vasculature. Furthermore, therapy might influence gene expression profiles in general. Preliminary data of the present study however could not observe such effect between B-cells from patients with and without epoprostenol treatment. The study of peripheral blood B-cell RNA expression profiles has further intrinsic limitation, as we do not know whether similar up-regulated transcripts would be found in the pulmonary vasculature itself. Finally, the biological significance of the genes detected has not been investigated by functional analyses. These issues will be addressed by subsequent studies. Despite these limitations, our studies suggest that B cells are activated in patient with IPAH. We strongly believe that the results present herein contribute significantly to our understanding of the pathogenesis of IPHA and thus might help to find new treatment strategies for this still incurable, devastating disease.

## Conclusion

We found that patients with IPAH express a distinct RNA expression profile in their peripheral blood B-lymphocytes that clearly suggests activation of B cells when compared with healthy controls The up-regulated transcripts herein described may help to direct future research on the pathogenesis of pulmonary arterial hypertension.

## Abbreviations

B-cells = B lymphocytes, IPAH = idiopathic pulmonary arterial hypertension, PBMC = peripheral blood mononuclear cells, RNA = ribonucleic acid

## Competing interests

The author(s) declare that they have no competing interests.

## Authors' contributions

SU, LTS and NV have made substantial contributions to conception and design, acquisition, analysis and interpretation of data. SU wrote the manuscript. LCH and RS have been involved in drafting the manuscript and revised it critically for important intellectual content, LTS and NV have given final approval of the version to be published. All authors read and approved the final manuscript.
